# Hypermethylated long noncoding RNA MEG3 promotes the progression of gastric cancer

**DOI:** 10.18632/aging.102309

**Published:** 2019-10-04

**Authors:** Lei Ding, Yuan Tian, Ling Wang, Miaomiao Bi, Dengke Teng, Sen Hong

**Affiliations:** 1 Department of Radiology, China-Japan Union Hospital of Jilin University, Changchun 130022, Jilin, China; 2 Department of Medical Examination, China-Japan Union Hospital of Jilin University, Changchun 130022, Jilin,China; 3 Department of Gynecology and Obstetrics, The Second Hospital of Jilin University, Changchun 130041, Jilin, China; 4 Department of Ophthalmology, China-Japan Union Hospital of Jilin University, Changchun 130022, Jilin, China; 5 Department of Ultrasonography, China-Japan Union Hospital of Jilin University, Changchun 130022, Jilin, China; 6 Department of Colorectal and Anal Surgery, The First Hospital of Jilin University, Changchun 130000, Jilin, China

**Keywords:** gastric cancer, methylation, MEG3, miR-181a-5p, ATP4B

## Abstract

This study aims to explore the expression and degree of methylation of lncRNA MEG3 in gastric cancer tissues and to analyze its effect on the migration and proliferation of gastric cancer patients and the mechanism by which this occurs. The targeting relationship between MEG3, miR-181a-5p and *ATP4B* was detected through molecular biology experiments. Wound healing, transwell, colony formation and flow cytometry assays were used to analyze the effects of lncRNA MEG3 and methylation on tumor cell migration, invasion, proliferation and apoptosis. In addition, a tumor xenotransplantation model was established to study the influence of MEG3 on tumor growth *in vivo*. Bioinformatics analysis showed that lncRNA MEG3 and *ATP4B* were downregulated in gastric cancer tissues compared with normal tissues. Bioinformatics predicted that *ATP4B* might be regulated by targeting miR-181a-5p. The overexpression of MEG3 and the application of 5-Aza treatment inhibited the migration, invasion and proliferation of MGC-803 cells and promoted apoptosis. In gastric cancer tissues, MEG3 is hypermethylated to decrease expression. Once the expression of MEG3 is restored or methylation is inhibited, tumor growth can be inhibited both *in vivo* and *in vitro*. This finding could be utilized as a clinical reference for gastric cancer treatment in the future.

## INTRODUCTION

As a typical malignancy, gastric cancer (GC) remains the third most widely common cancer and has resulted in an increasing huge number of deaths worldwide [1, 2]. Since metastasis, which is a complicated pathological procedure that is responsible for most cancer-related mortalities, has already happened once vague symptoms are detected, the prognosis and treatment of GC patients is still far from satisfactory [2, 3]. GC is the third leading cause of cancer-related death in Asia. As the Asian population is aging, the number of elderly patients with this disease is increasing [4]. Due to this fact, a more comprehensive and profound understanding of GC is urgently needed to discover an optimized and efficient diagnostic strategy to prevent and treat gastric cancer at an early stage [3].

DNA methylation is widely studied in various fields of diseases. It regulates the expression of various genes, especially those related to cancer, and tumors usually reflect distinctive patterns of DNA methylation [5]. Several cancers show methylations of various genes in distinct tissues, and some of them are age- related methylations [6]. Among all these cancers, GC is a dominant epigenetic phenomenon that depends greatly on a changed DNA methylation state [7]. In addition, the abundance of abnormal DNA methylation in gastric mucosae, mostly induced by *H. pylori* infection, is also strongly related to a predisposition to gastric cancers [8].

The compound 5-Aza-2-deoxycytidine is a demethylating agent that causes genome-wide hypomethylation and inhibits cancer cell growth [9]. It plays a dominant role in the treatment of myelodysplastic syndrome and acute myeloid leukemia (AML) [10]. In most GC cells, HHIP promoter methylation and cancer cell proliferation were both suppressed by 5-Aza-dc [11]. Furthermore, 5-Aza could also be utilized to analyze the differential expression of lncRNAs in gastrointestinal cancer cells and upregulated most lncRNAs, showing that these lncRNA expression levels might be associated with DNA methylation [12].

Among most tumor-associated lncRNAs, the lncRNA MEG3 (maternally expressed gene 3) has attracted much attention. Research by Sun suggested that MEG3 overexpression inhibited endometrial cancer cell proliferation, invasion, and metastasis through the PI3K pathway [13]. In addition, the role of MEG3 in GC has also attracted’ attention. Through *in vivo* and *in vitro* binding experiments, Dan et al. verified that MEG3 may affect the occurrence and development of gastric cancer by affecting the expression of miR-21 [14]. Studies on MEG3 in patients with diabetes have shown that DNA methylation may occur in the promoter region, resulting in decreased expression of MEG3 and affecting the occurrence and development of the disease [15, 16]. However, whether it can play a regulatory role in gastric cancer through DNA methylation has not been studied. Therefore, the methylation level and its regulatory mechanism in gastric cancer are the focus of this study.

Apart from MEG3, microRNA-181a-5p (miR-181a-5p) has an indispensable role in GC patients as well. It serves as a tumor suppressor regulating target genes at the posttranscriptional stage [17]. It is also an upstream regulator of Egr1, which is downregulated in the TIF of diabetic nephropathy and increased in HK-2 cells treated with high glucose [18]. By negatively targeting INPP5A, the overexpression of miR-181a-5p could suppress apoptosis of cervical cancer cells [19]. MiR-181a-5p is also upregulated in osteosarcoma, and this overexpression benefits the proliferation and suppresses the apoptosis of osteosarcoma [20].

Another crucial gene, *ATP4B*, always serves as a desirable aim for acid reduction because it regulates the secretion of gastric acid [21]. Its expression was restored by 5-Aza or TSA in gastric cancer cell lines but facilitated chemotherapeutic mediation with docetaxel to inhibit GC cell growth [21]. Studies have shown that *ATP4B* is expressed at low levels in gastric cancer tissues compared with normal tissues. In addition, studies have explored the possibility of *ATP4B* as a biomarker for gastric cancer screening, verifying the possibility of early diagnosis of gastric cancer through screening of plasma expression [22, 23]. However, its upstream regulatory mechanism in GC is still unclear.

In this study, we first analyzed the relationship between MEG3 and *ATP4B* through bioinformatics analysis. The regulatory mechanism is discussed through database prediction. We aim to explore the influence of epigenetic regulation of MEG3 on tumor cell migration, invasion, proliferation and apoptosis and to study the previous targeting relationship among these three functions and the influence of MEG3 reintroduction on tumor growth.

## RESULTS

### LncRNA MEG3 and *ATP4B* were downregulated in gastric cancer tissues

**Figure 1:**
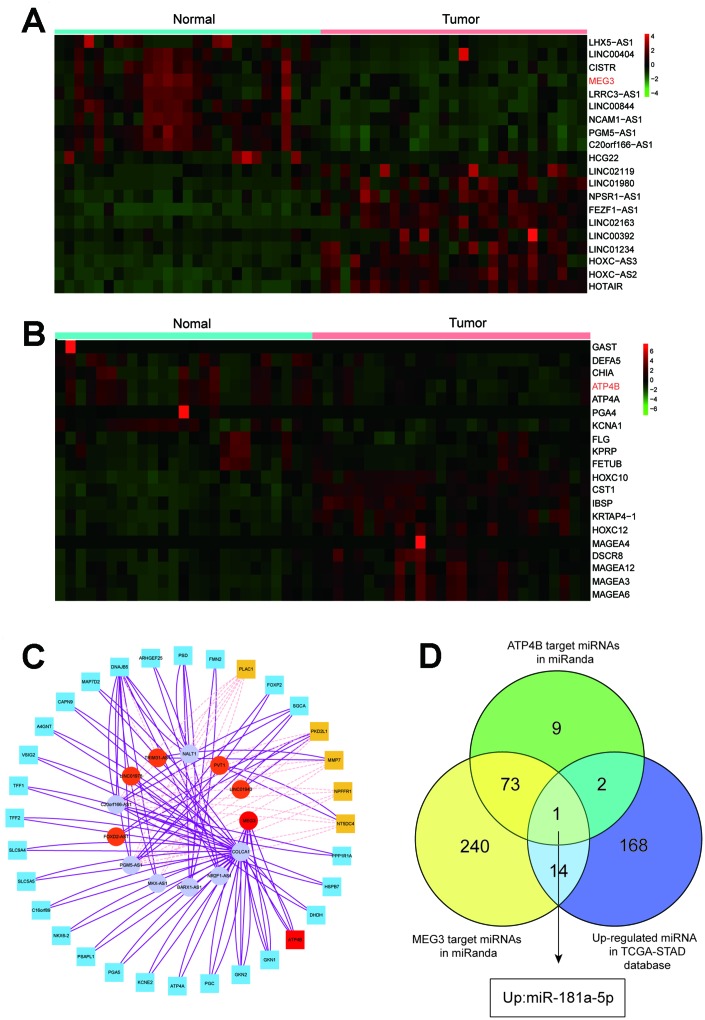
**Heat map analysis and target gene prediction.** (**A**) Heat map analysis showed that MEG3 was downregulated among the top 20 differentially expressed lncRNAs in GC tissues and adjacent normal tissues. (**B**) Heat map analysis showed that the expression of *ATP4B* was reduced among the top 20 differentially expressed mRNAs between GC tissues and adjacent normal tissues. (**C**) The network of the relationships between the top twelve differentially expressed genes in lncRNA and the top thirty differentially expressed genes in mRNA. (**D**) MEG3 and *ATP4B* are highlighted in a Venn analysis of miRNA associated with gastric cancer from the TCGA database.

Heat map analysis showed that MEG3 was downregulated among the top 20 differentially expressed lncRNAs in GC and adjacent normal tissues (Figure 1A). Additionally, *ATP4B* expression was reduced among the top 20 differentially expressed mRNAs between these tissues (Figure 1B). Expression values are represented in shades of red and green, which indicate high expression levels and low expression levels, respectively. All the results suggested that MEG3 and *ATP4B* were downregulated in GC patients.

WGCNA was used to detect gene expression profiles, and the gene coexpression modules were constructed. Coexpression clusters for normal and tumor tissues were detected by hierarchical cluster analysis (Supplementary Figure 1), and each vertical line represents one gene. Supplementary Figure 1 shows the module details of normal tissues. Supplementary Figure 2 displays the size of each module for GC tissues, and we can deduce the correlation between the sample trait and module eigengene. A total of 9 gene modules are shown (Supplementary Figure 2); the genes marked red are hub genes in GC tissues. These genes are *ADAMTS2*, *LMX1A*, *MYH11*, *TAGLN*, *ZG16*, *ATP4B*, *RBP2*, *MAGEA4*, and *IBSP* for red, blue, pink, purple, yellow, black, brown, green and magenta modules, respectively. The Y-axis value represents the significance of the gene in each module, and the X-axis value represents the correlation between the module and gene. The *ATP4B* module in black was chosen for the following analysis.

A network was constructed of the relationships between the top twelve differentially expressed genes in the lncRNA analysis and the top thirty differentially expressed genes in the mRNA analysis, indicating that they were in contact with each other (Figure 1C). The Venn diagram analysis showed that miR-181a-5p was most correlated with MEG3 and *ATP4B* from the TCGA data (Figure 1D). Among them, miR-181a-5p has the strongest relationship with GC, so it is the target of further study.

### Clinical significance of lncRNA MEG3 expression in GC tissues

We further explored the relationship between MEG3 expression and clinicopathological factors. The MEG3 expression level was correlated with TNM staging, lymph node metastasis and tumor size (Supplementary Table 1) but not with age, sex, extrathyroidal extension, or multicentricity of the clinical pathological characteristics examined.

### MEG3 was hypermethylated and expressed at low levels in GC tissues and cells

**Figure 2:**
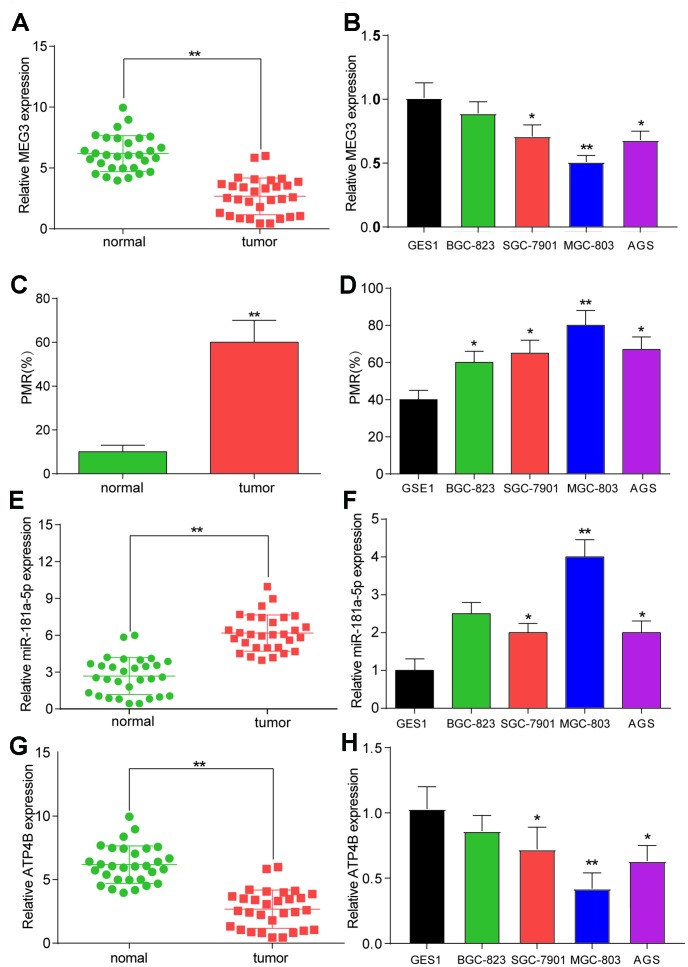
**The expression and methylation status of target factors in gastric cancer and gastric cancer cell lines.** (**A**) LncRNA MEG3 was found to be expressed at low levels in thirty GC samples compared to normal tissues. **P<0.01, compared with 30 matched normal tissues. (**B**) LncRNA MEG3 was also found to be downregulated in BGC-823, SGC-7910, MGC-803 and AGS GC cell lines compared to the GES 1 normal cell line. *P<0.05, **P<0.01, compared with the GES 1 normal cell line. (**C**) MSP results showed that lncRNA MEG3 was hypermethylated in tumor tissues compared with normal tissues. **P<0.01, compared with 30 matched normal tissues. (**D**) Hypermethylation of lncRNA MEG3 was also detected in BGC-823, SGC-7910, MGC-803 and AGS GC cell lines compared to the GES 1 normal cell line. *P<0.05, **P<0.01, compared with the GES 1 normal cell line. (**E**) MiR-181a-5p was upregulated in tumor tissues compared with their corresponding normal tissues. **P<0.01, compared with 30 matched normal tissues. (**F**) QRT-PCR results showed higher expression of miR-181a-5p in BGC-823, SGC-7910, MGC-803 and AGS GC cell lines compared to the GES 1 normal cell line. *P<0.05, **P<0.01, compared with the GES 1 normal cell line. (**G**) QRT-PCR results showed lower expression of *ATP4B* in tumor tissues compared to normal tissues. **P<0.01, compared with 30 matched normal tissues. (**H**) QRT-PCR results showed higher expression of ATP4B in BGC-823, SGC-7910, MGC-803 and AGS GC cell lines compared to the GES 1 normal cell line. *P<0.05, **P<0.01, compared with the GES 1 normal cell line.

LncRNA MEG3 was found to be expressed at low levels in thirty GC samples compared to normal tissues (Figure 2A, *P*<0.01). LncRNA MEG3 was also found to be downregulated in BGC-823, SGC-7910, MGC-803 and AGS GC cell lines, compared to the GES 1 normal cell line (Figure 2B). The MSP results showed that lncRNA MEG3 was hypermethylated in tumor tissues compared with normal tissues (Figure 2C, *P*<0.01). Hypermethylation of lncRNA MEG3 was also detected in BGC-823, SGC-7910, MGC-803 and AGS GC cell lines compared to the GES 1 normal cell line (Figure 2D). Since these changes were most significant in MGC-803 cells, this cell line was selected for the following analysis.

### The expression of *ATP4B* and miR-181a-5p in GC tissues and cells

MiR-181a-5p was found to be upregulated in tumor tissues compared with their corresponding normal tissues (Figure 2E, *P*<0.01). QRT-PCR results exhibited higher expression of miR-181a-5p in BGC-823, SGC-7910, MGC-803 and AGS GC cell lines than in the GES 1 normal cell line (Figure 2F). These findings suggested that miR-181a-5p was upregulated in GC patients. In addition, qRT-PCR results showed lower expression of *ATP4B* in tumor tissues compared to normal tissues (Figure 2G, *P*<0.01). *ATP4B* was also downregulated in BGC-823, SGC-7910, MGC-803 and AGS GC cell lines compared to the GES 1 normal cell line (Figure 2H, *P*<0.01).

### The target verification of lncRNA MEG3, miR-181a-5p and *ATP4B*

**Figure 3:**
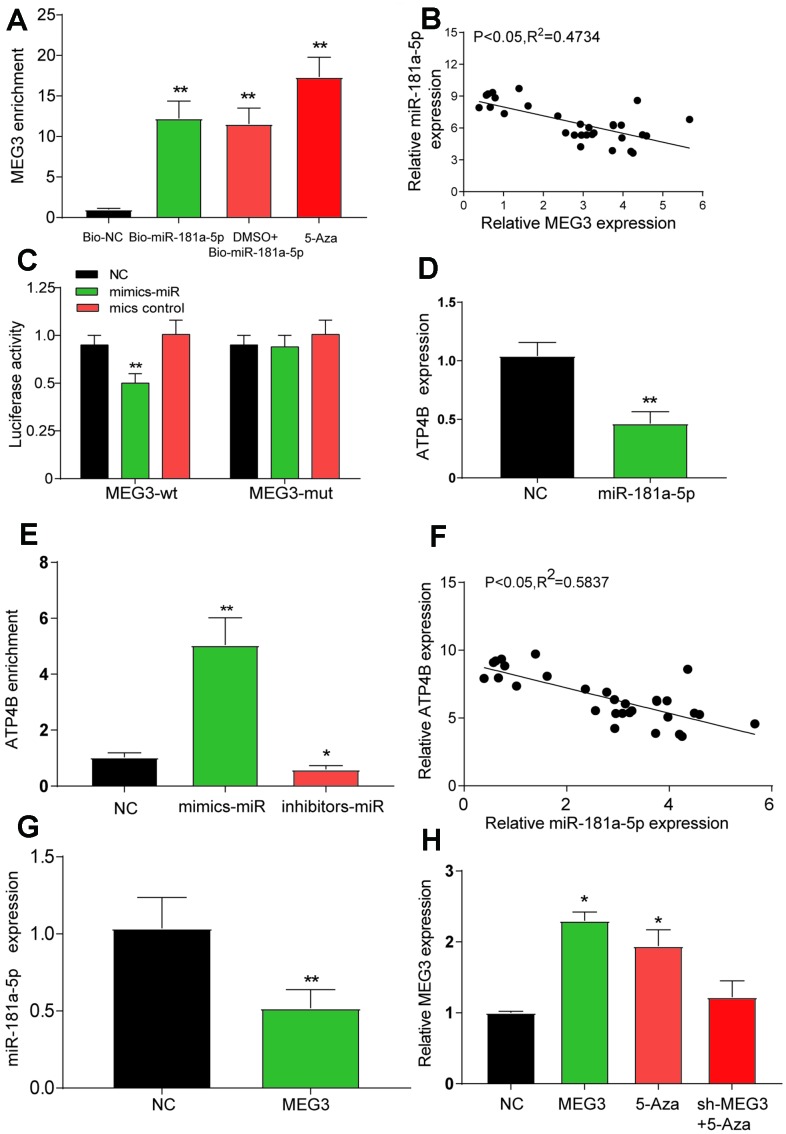
**Verification of the target relationship between the three target genes.** (**A**) The RNA pull-down test showed a low level of MEG3 enrichment in the bio-NC group than in the bio-miR-181a-5p, DMSO + 5-Aza and 5-Aza treatment groups. **P<0.01, compared with the bio-NC group. (**B**) A negative correlation between the expression of MEG3 and miR-181a-5p was detected by linear regression analysis after qRT-PCR detection of 30 tissue specimens. (**C**) Luciferase reporter assay showed lower luciferase activity in the mimic-miR + lncRNA MEG3-wt group than in the mimic-miR + lncRNA MEG3-mut group. *P<0.05, **P<0.01, compared with the NC group. (**D**) The expression of *ATP4B* was verified by qRT-PCR after the overexpression of miR-181a-5p. **P<0.01, compared with the NC group. (**E**) The RNA pull-down test showed a higher level of *ATP4B* enrichment in the inhibitor-miR group than in the mimic-miR group*.* *P<0.05, compared with the NC group. (**F**) qRT-PCR detection showed a negative relationship between the expression of *ATP4B* and miR-181a-5p*.* (**G**) The expression of miR-181a-5p was verified by qRT-PCR after the overexpression of MEG3. *P<0.05, compared with the NC group. (**H**) The expression level of each treatment group MEG3. *P<0.05, compared with the NC group.

The luciferase reporter assay showed that the luciferase activity in the MEG3-wt group mimics + miR was lower than that in the NC group (Figure 3C, *P*<0.01). RNA pull-down experiments showed that MEG3 enrichment levels in the bio-miR-181a-5p, DMSO + bio-miR-181a-5p and 5-Aza groups were higher than those in the bio-NC group (Figure 3A, *P*<0.01). In Figure 3B, linear regression analysis showed that the expression of MEG3 was negatively correlated with the expression of miR-181a-5p. As shown in Figure 3G, the expression of miR-181a-5p in the MEG3 overexpression group was significantly lower than that in the NC group (*P*<0.01). In addition, the enrichment level of *ATP4B* in the inhibitor - miR group was higher than that that in the mimics - miR group (Figure 3E, *P*<0.05). Linear regression analysis by qRT-PCR showed that the expression of *ATP4B* was negatively correlated with miR-181a-5p (Figure 3F), and the expression level of *ATP4B* in the miR-181a-5p overexpression group was significantly lower than that in the NC group (Figure 3D). All the results showed that *ATP4B* and miR-181a-5p were negatively regulated.

### The restored expression of MEG3 inhibited the progression of GC cells

**Figure 4:**
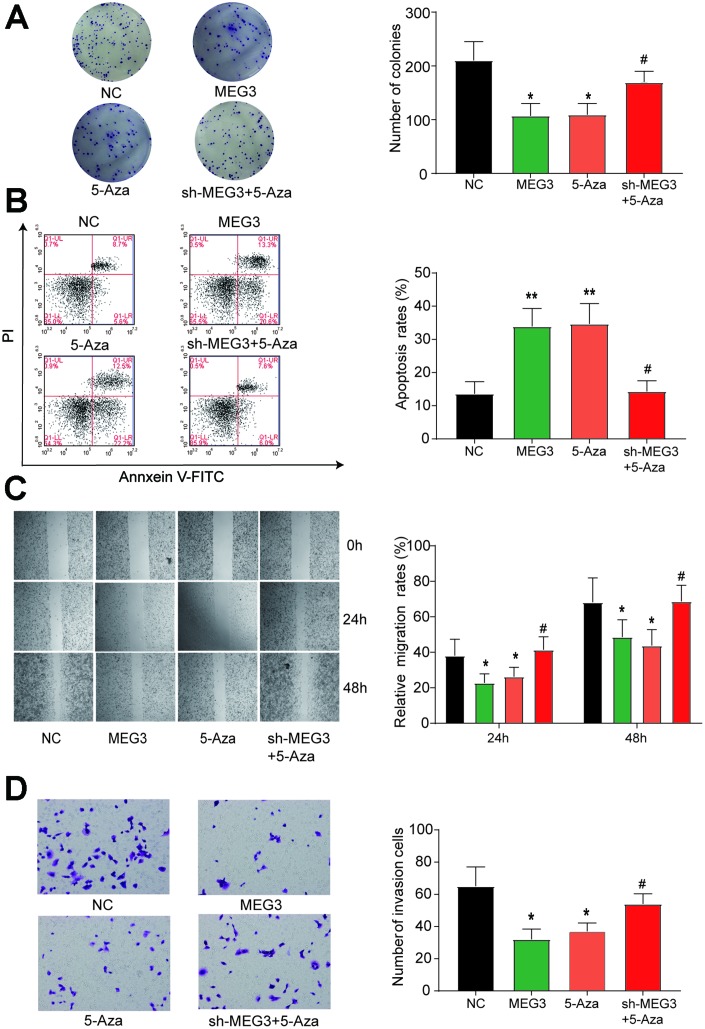
**Restored expression of MEG3 inhibits the development of gastric cancer cells.** (**A**) The number of clones was significantly reduced in the lncRNA MEG3-overexpressing group and the 5-Aza group compared with the NC group. *P<0.05, **P<0.01, compared with the NC group. #*P*<0.05, compared with the 5-Aza group. (**B**) The apoptosis rate of MGC-803 cells transfected with MEG3 or 5-Aza dramatically increased compared with the NC group. *P<0.05, **P<0.01, compared with the NC group. #*P*<0.05, compared with the 5-Aza group. (**C**–**D**) Wound healing and transwell assays showed significantly reduced cell migration and invasion ability in the MEG3 and 5-Aza groups compared to the control group. *P<0.05, **P<0.01, compared with the NC group. #*P*<0.05, compared with the 5-Aza group.

We verified the expression levels of MEG3 in each treatment group and found significant increases in the overexpression group and the 5-Aza treatment group (Figure 3H). The number of clones was significantly reduced in the lncRNA MEG3 overexpression group and the 5-Aza group compared with the NC and sh-MEG3 + 5-Aza groups (Figure 4A). The apoptosis rate of MGC-803 cells transfected with MEG3 or 5-Aza dramatically increased compared with the NC and sh-MEG3 + 5-Aza groups (Figure 4B, *P*<0.05). Wound healing and transwell assays showed significantly reduced migration and invasion ability of cells in the MEG3 and 5-Aza groups relative to the NC and sh-MEG3 + 5-Aza groups (Figure 4C–4D). When we combined all the data, we concluded that MEG3 served as a tumor inhibitor gene suppressing the migration, invasion, and proliferation of GC cells while promoting apoptosis.

### Overexpression of MEG3 inhibits tumor growth *in vivo*

**Figure 5:**
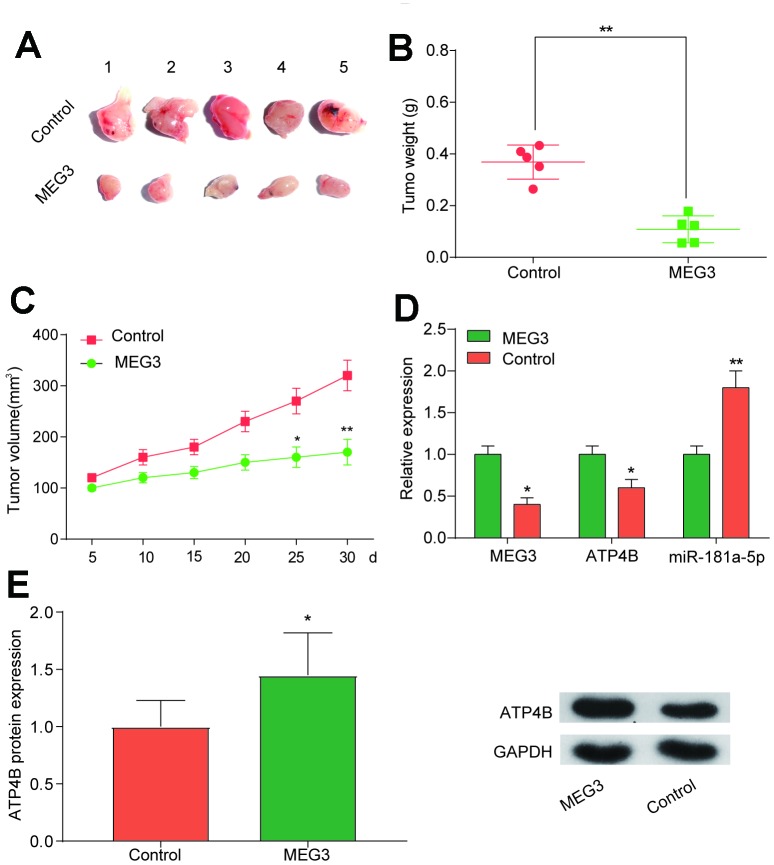
**Overexpression of MEG3 *in vivo* inhibits tumor growth.** (**A**–**C**) Tumor xenograft results showed that tumor weight and volume were decreased in the MEG3 group compared with the Con group. *P<0.05, **P<0.01, compared with Con group. (**D**) QRT-PCR results illustrated that *ATP4B* expression decreased while miR-181a-5p expression increased in the MEG3 group compared with the Con group. (**E**) Western blotting results illustrated that ATP4B expression increased in the MEG3 group compared with the Con group. *P<0.05, **P<0.01, compared with Con group.

To investigate the effect of MEG3 on tumor growth, we transfected MGC-803 cells with MEG3 to observe tumor size and volume. The results showed that tumor weight and volume in the MEG3 group were lower than those in the Con group (Figure 5A–5C, *P*<0.01). Next, we studied the expression of *ATP4B* and miR-181a-5p after MEG3 transfection. The results showed that the expression of *ATP4B* was increased in the MEG3 group, while the expression of miR-181a-5p was decreased (Figure 5D–5E, *P*<0.01). Therefore, we demonstrated a negative correlation between MEG3 and miR-181a-5p and found that upregulation of MEG3 *in vivo* can inhibit tumor growth.

## DISCUSSION

In this paper, the differentially expressed lncRNAs and mRNAs in gastric cancer patients were analyzed by using data from the TCGA database, and MEG3 and *ATP4B* were significantly downregulated. In addition, *ATP4B* was found to have the highest correlation with GC by WGCNA analysis. Network analysis showed that *ATP4B* and MEG3 have the same expression trend in gastric cancer and are closely related. Therefore, MEG3 and *ATP4B* were selected for further study. We analyzed the differentially expressed miRNAs from the TCGA database, analyzed the miRNA with the predicted targeting of *ATP4B* and MEG3 by the Venn diagram method, and found seven miRNAs with targeting relationships. First, the preliminary screening of upregulated miRNAs revealed that miR-181a-5p is the most closely related with the same trend in gastric cancer. Therefore, this study selected miR-181a-5p for further study [24–26]. We have established *in vitro* and *in vivo* research models to validate the targeting of MEG3, miR-181a-5p and *ATP4B* and their regulatory mechanisms for GC.

Studies have shown that the expression of MEG3 is downregulated in GC tissues and can regulate the proliferation and apoptosis of gastric cancer cells by targeting the expression of miR-21 [14]. The mechanism by which the expression of MEG3 is reduced is unclear. Predecessors in the study of diabetes found that the promoter region of MEG3 can undergo DNA methylation and that DNA methylation can reduce the expression of target genes [15, 16]. Numerous studies have shown that DNA methylation levels increase to varying degrees in most tumor patients [27–29]. Therefore, this study sought to investigate whether the mechanism of downregulation of MEG3 in gastric cancer is related to DNA methylation and the degree of methylation in gastric cancer patients. From the results of this study, we can see that the expression level of MEG3 in gastric cancer patients is significantly lower than that in adjacent tissues, which is consistent with the results of previous studies. Further detection of its methylation level revealed a significant increase in the methylation level of MEG3 in GC tissues, and this phenomenon was also verified in multiple GC cell lines. Therefore, we can see that DNA methylation may affect the development of GC by affecting the expression of MEG3. The specific regulatory mechanism in between is still of interest to us.

In this study, bioinformatics prediction combined with literature screening of ATP4B and miR-181a-5p suggested that they may serve as downstream regulators of MEG3 to exert its biological effects. First, we validated our predictions in cancer tissues and found that miR-181a-5p was upregulated and *ATP4B* expression was downregulated in GC tissues. This result is consistent with those of previous studies. Studies have shown that miR-181a-5p is upregulated in gastric cancer patients and that *ATP4B* is downregulated in the plasma of gastric cancer patients and could act as a biomarker. This possibility was explored [22, 26]. However, in this study, the targeting relationship of these three components was verified by bioinformatics prediction and molecular biology. From the dual luciferase reporter and RNA pull-down experiments, we can see that MEG3 can target miR-181a-5p and that overexpression of MEG3 leads to a significant decrease in the expression level of miR-181a-5p. At present, it has been confirmed that MEG2 can affect the occurrence of gastric cancer by targeting miR-181a-5p [25], and the targeting of miR-181a-5p by its family member MEG3 is also verified in this paper. Similarly, in the targeted validation of *ATP4B* and miR-181a-5p*,* we obtained confirmation via RNA pull-down and overexpression experiments. In summary, we can clearly see that MEG3 targets miR-181a-5p and thus affects the regulatory mechanism of *ATP4B*.

Further, this study investigated the biological effects of these three components in gastric cancer. In the results of *in vitro* experiments, we can see that the MEG3 overexpression group and 5-Aza treatment group have a significant effect on cell proliferation, apoptosis, migration and invasion. In addition, we added the 5-Aza + sh-MEG3 treatment group to further demonstrate that the growth of gastric cancer after DNA methylation inhibition was not significantly improved compared with the *MEG3* overexpression and 5-Aza treatment groups. The increase is because 5-Aza, as a global methylation inhibitor, may differentially inhibit methylation levels [30]. This is also one of the limitations of the current study. However, through our experimental design grouping, it can be seen that DNA methylation regulates the expression of MEG3 and affects miR-181a-5p and its downstream target gene *ATP4B*. Formal DNA methylation can exert biological effects by affecting the expression of lncRNAs and affecting downstream target genes [31]. Other studies have shown that DNA methylation affects the expression of MEG3 and affects its targeting of miR-29 in liver cancer [32]. This study validated a similar mechanism in GC.

Despite the significant findings and profound conclusions, we acknowledge that there are some limitations remaining in this study: the number of samples might not be adequate enough and the possibility of other underlying factors might not be totally excluded. However, we still carried out comprehensive analysis and robust models to determine the methylation levels of MEG3 and miR-181a-5p and their impacts on GC cells Since most of these genes studied in our research could serve as biomarkers for GC, further investigation is urgent for GC diagnosis and treatment.

## MATERIALS AND METHODS

### Clinical samples

According to previous studies, we made reference to the selection of case samples, and the selected tissue samples were chosen by blind selection of two pathologists for inclusion in this study. Finally, we determined 30 patients as the sample number of this study [25, 33, 34]. A total of 30 paired tumor and normal tissues were obtained from GC patients who were undergoing treatment at the First Hospital of Jilin University (Jilin, China). The corresponding noncancerous tissues were at least 5 cm away from the tumor edge. The specimens were snap-frozen in liquid nitrogen and stored at a temperature of -80°C for molecular analyses. All subjects gave informed consent for obtaining the study materials. The study protocol was agreed by the Clinical Research Ethics Committee of the First Hospital of Jilin University (Jilin, China).

### Cell culture

Four human gastric cancer cell lines (BGC-823, SGC-7910, MGC-803 and AGS) and a normal human gastric mucosa cell line (GES 1) were received from BeNa Culture Collection (Beijing, China). Cells were kept at 37°C in a humid atmosphere with 5% CO_2_. BGC-823 cells were cultured in Roswell Park Memorial Institute (RPMI) medium (Sigma-Aldrich, St. Louis, MO, USA) containing 10% FBS (Invitrogen, CA, USA). SGC-7910 cells were cultured in Roswell Park Memorial Institute 1640 (RPMI1640) medium (Sigma-Aldrich, St. Louis, MO, USA) with 10% FBS (Invitrogen, CA, USA). MGC-803 cells were maintained in Dulbecco’s Modified Eagle’s Medium (DMEM – 214.5 g/L glucose) containing 10% FBS (Invitrogen, Carlsbad, CA, USA). AGS cells were cultured in Nutrient Mixture F-12 (F-12) (Sigma-Aldrich, St. Louis, MO, USA) supplemented with 10% FBS (Invitrogen). GES 1 cells were cultured in RPMI medium (Sigma-Aldrich, St. Louis, MO, USA) containing 10% FBS (Invitrogen).

### Methylation-specific PCR (MSP)

Bisulfite-converted DNA (~50 ng) was amplified in a one-step MSP reaction with 0.2 μM of each deoxynucleotide triphosphate and primer (both methylated and unmethylated forward and reverse primers) using 1 U Maxima® Hot Start Taq DNA polymerase (Thermo Fisher Scientific, Inc.). The respective PCR conditions were as follows: one cycle at 98°C for 30 seconds (initial denaturation); 35 cycles at 98°C for 10 seconds (denaturation), 62°C for 30 seconds (annealing) and 72°C for 30 seconds (extension); and one cycle at 72°C for 7 minutes (final extension). MSP validity was hindered by the amplitude of CpG-methylated human genomic DNA acting as the negative control; however, for the non-methylation-specific PCR, human genomic DNA that was not converted by bisulfite was utilized as the positive control. PCR procedures were performed with a BIOER™ Thermal Cycler 9500 (Hangzhou Bioer Technology Co., Ltd., Binjiang, China). The percentage of methylated reference (PMR) was calculated as the fraction of the methylated reference [(methylated molecules in a sample)/(total DNA molecules in a sample)]/[(methylated molecules in SssI-treated DNA)/(total DNA molecules in SssI-treated DNA)] [35]. All of the primers involved were purchased from AuGCT Biotechnologies (Beijing, China), and primer sequences are shown in Supplementary Table 2.

### Cell transfection

To enhance the exogenous expression of MEG3, the full length human MEG3 was synthesized by PCR according to the GenBank gene sequence (NR_002766) and inserted into the pcDNA3.1 empty plasmid (GenePharma, Shanghai, China); Control (pcDNA3.1 vector) was used for comparison [14]. To reduce the expression of MEG3, GenePharma synthesized shRNA (MEG3 shRNA) against MEG3. The sequence of the MEG3 shRNA is as follows: 5′-GAGAGGTTGTTTCACTGGTATCTATTG CA-3′. MGC-803 cells were seeded in 96-well plates and cultured to 60% confluency for transfection. Transfection was performed using Lipofectamine 2000 (Thermo Fisher Scientific) according to the manufacturer’s instructions. The concentrations of pcDNA3.1-MEG3 and MEG3 shRNA for transfections were 100 nM.

### Demethylation with 5-Aza-dC treatment

Cells were seeded at a density of 1 × 10^6^ cells/ml. After 24 hours, cells were treated with 1 μM 5-Aza-dC (Sigma-Aldrich, St. Louis, MO, USA) and an equivalent concentration of vehicle (dimethyl sulfoxide) for 5 days and then were harvested for DNA and RNA extractions.

### Luciferase reporter assay

The 3′UTR fragment of lncRNA *MEG3* was amplified and replicated into the PmeI and XbaI sites of the pmirGLO vector (Promega, Madison, WI, USA). The mutant MEG3 3′UTR fragment was also developed using site-directed mutagenesis. According to reporter assays, cells were formed in 24-well plates and transfected with MEG3-wt or MEG3-mut type luciferase reporters and then cotransfected with miR-181a-5p mimics or mimics control. Each assay was performed three times. Treated cells were analyzed for luciferase activity with the Dual-Glo Luciferase Assay System (Promega, WI, USA) and a MicroLumatPlus LB96V luminometer (Berthold, USA) after 48 hours.

### RNA pull-down test

Biotinylated miR-181a-5p, biotinylated mutant, and biotinylated NC were synthesized by GenePharma (Shanghai, China). The biotinylated miRNA was transfected into MGC-803 cells. The cell lysates were incubated with M-280 streptavidin magnetic beads (Invitrogen, USA). RT–qPCR was employed to probe the levels of *MEG3*.

### QRT-PCR

RNA from cells or tissue samples was separated with the mirVana miRNA Isolation Kit (Ambion, Austin, TX, USA) and reverse transcribed to cDNA with the First Strand cDNA Synthesis Kit (Fermentas, Burlington, Canada). These cDNAs were transferred to qPCR with the SYBR premix ExTaq kit (TaKaRa, Dalian, China) to probe the expression of lncRNA *MEG3*, *ATP4B* or miR-129-5p. For lncRNA *MEG3* and *ATP4B* expression, paired primers (forward and reverse) were utilized, and glyceraldehyde phosphate dehydrogenase (GAPDH) acted as the endogenous reference gene. For miR-181a-5p expression, paired primers (forward and reverse) were utilized with U6 snRNA for normalization. All of the reactions were carried out with an Applied Rotor-Gene 6000 Real Time machine. The relative expression was calculated using the $2^{-\Delta \Delta C_{t}}$ method [36]. All of the primers involved were purchased from AuGCT Biotechnologies (Beijing, China) and are shown in Supplementary Table 2.

### Transwell invasion assay

Eight-micrometer pore polycarbonate membranes (BD Bioscience) coated with 25 μg Matrigel® (BD Biosciences) were applied in transwell chambers. A total of 5 × 10^4^ cells in a medium containing 0.5-1% FBS was placed in the upper chamber, while 5-10% FBS medium was placed in the lower chamber. Non-migrated cells were removed with a cotton swab while migrated cells were fixed with 4% paraformaldehyde and stained with 0.5% crystal violet after 24 hours. The total number of migrated cells was counted and photographed with a Nikon Eclipse E600 microscope (Nikon Instruments, Japan).

### Clone formation assay

A total of 5 × 10^3^ cells were seeded into six-well culture plates and replenished every 3 days with complete medium. Cells were subsequently fixed with methanol and 0.5% crystal violet. Visible colonies were manually counted in randomly selected fields with a Nikon Eclipse E600 microscope (Nikon Instruments, Melville, New York, USA). The clone formation rate (CFR) was computed according to the following formula: CFR = clone counts/seeded cell counts ×100%. The experiment was repeated 3 times.

### Flow cytometry analysis

Transfected MGC-803 cells were washed and resuspended. Immunofluorescence staining was performed at 4°C with the construction of the Annexin V-FITC Apoptosis Staining/Detection kit (Cambridge, MA, USA). After cleaning with FACS buffer, cells were delivered to conduct multichannel analysis by a FACScan flow cytometer (BD Biosciences, San Jose, CA, USA) and were then analyzed with Cell-Quest 3.3 software (BD Biosciences).

### Wound healing assay

Transfected cells were seeded into 6-well plates at 1 × 10^5^ cells/l. A 10 μl Eppendorf tip was used to scratch the center of the plate. The cell movement was recorded at 0, 24 and 48 hours by Image-Pro Plus 6.0 software. The migration ability was computed as follows: (prime scratch width ‒ current scratch width)/prime scratch width. The experiment was repeated 3 times.

### Tumor xenograft

Experimental animals (4-week-old female BALB/c nude mice) were purchased from the Animal Center of the First Hospital of Jilin University (Jilin, China). For epigenetic therapy, MGC-803 cells (1× 10^6^/200 μl PBS) were subcutaneously inoculated into the right flank of BALB/c nude mice. When the average tumor size reached approximately 100 mm^3^, the animals were separated into 2 groups. The epigenetic therapy used in this study was the sh*-MEG3* group (5 mg/kg) and the sh-Con group. Tumor diameters were measured with a Vernier caliper, and mice were evaluated every five days.

### Weighted gene coexpression network analysis

The coexpression network module is identified using the average RPM value in R and the WGCNA package (v3.4.1) [37]. Genes with a lower mean RPM coefficient of variation (CV < 1) in all sample types (cell/tissue type, different latitude sections or developmental stages) were excluded, and the remaining genes were used for analysis. The average relative RPM value summarizes the expression patterns of each module. The module characteristic gene (ME) value for each module is calculated, which summarizes the expression profile of a given module as the first principal component. The intermodule similarity is calculated as the PCC between the ME values. The signed KME algorithm was used to calculate the PCC between each gene RPM expression level and the module ME, and the ME-based gene connectivity (kME) was calculated, which is a quantitative factor indicating the correlation intensity of individual genes in each module. Package WGCNA_3.4.1 was used in this study to screen the hub gene in each gene module [38].

### Statistical analysis

Statistical analysis was conducted with GraphPad Prism 6.0 Software (GraphPad Inc., La Jolla, CA, USA). All of the experiments were repeated at least three times. Differences in expression among groups were tested using one-way analysis of variance (ANOVA). The chi-square test was used to stratify and compare clinical samples. A value of *P* < 0.05 was regarded as statistically significant.

## Supplementary Material

Supplementary Figures

Supplementary Tables
