# Supplementary material for: Hypermethylated long noncoding RNA MEG3 promotes the progression of gastric cancer
Source: Aging (Albany NY). 2019 Oct 4;11(19):8139–55. doi: 10.18632/aging.102309 (PMC6814614; doi:10.18632/aging.102309)
Supplement: Supplementary Figures [file aging-11-102309-s002.pdf]

SUPPLEMENTARY FIGURES

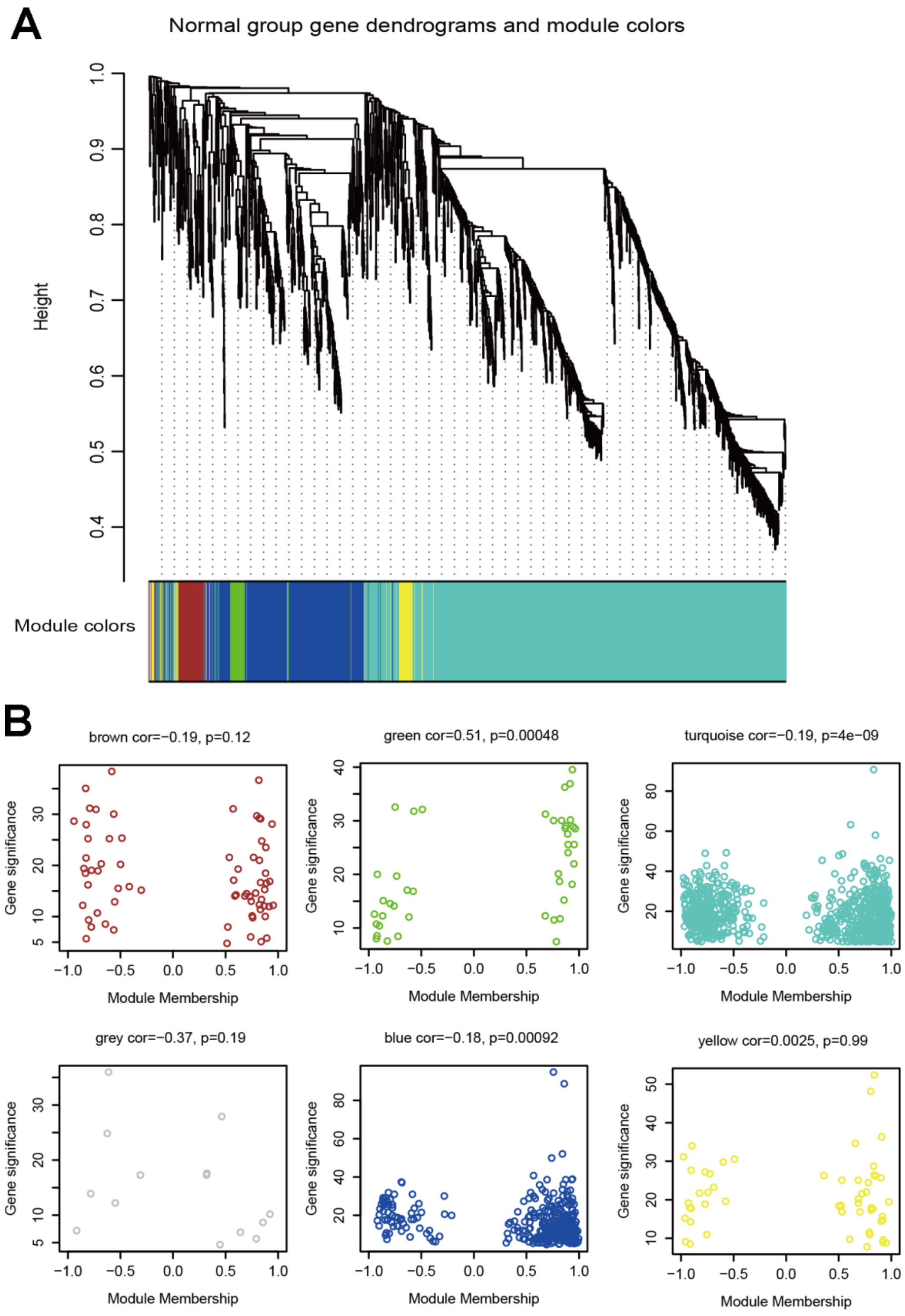

Supplementary Figure 1. WGCNA analysis for normal tissues. (**A**) Hierarchical cluster analysis showed coexpression clusters for normal tissues. (**B**) The details of each module for the normal group.

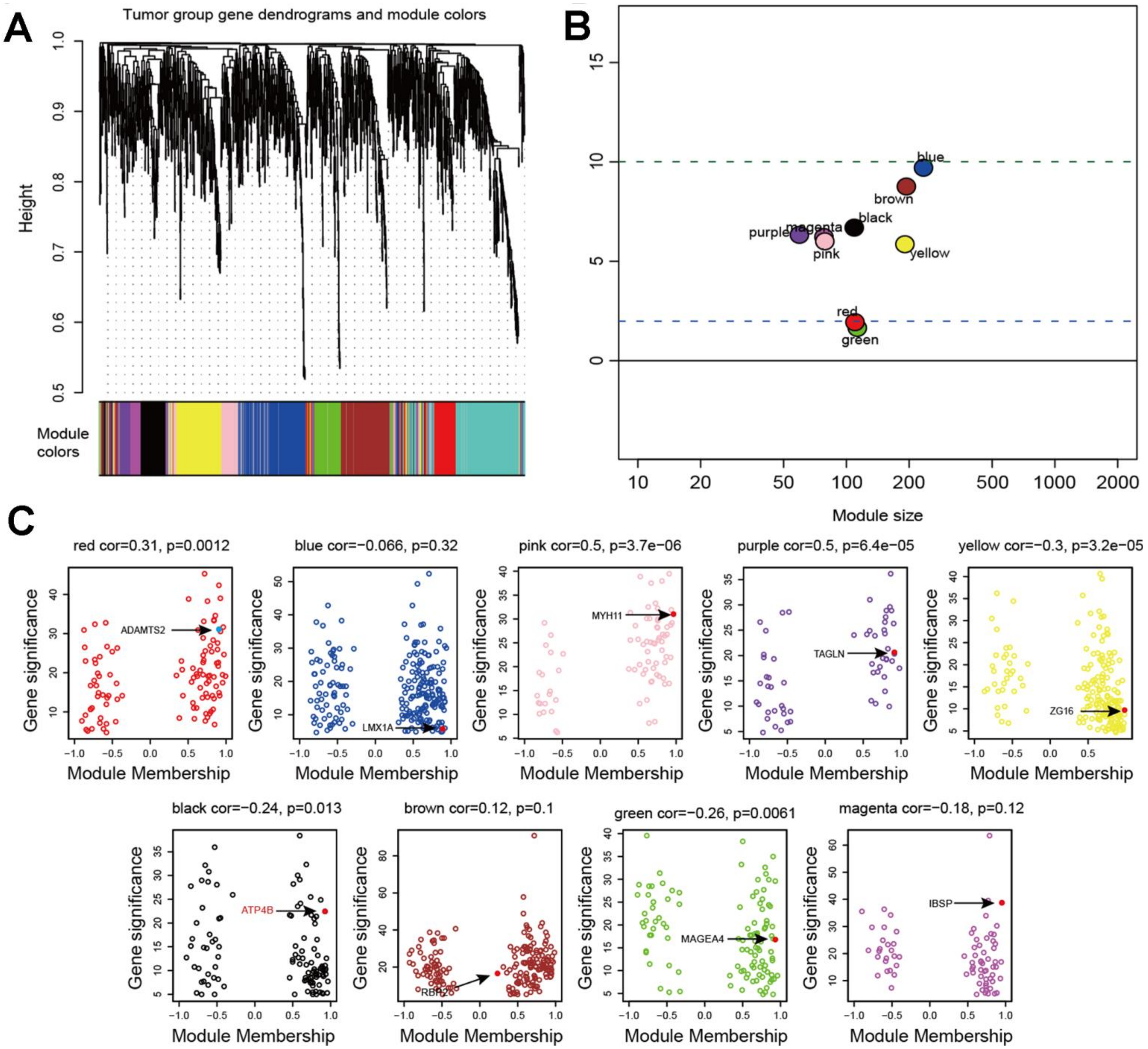

Supplementary Figure 2. WGCNA analysis for tumor tissues. (**A**) Hierarchical cluster analysis showed coexpression clusters for tumor tissues. (**B**) The size of each module for GC tissues. (**C**) The details of each module for the tumor group and marked hub gene for each module.
