# Supplementary material for: Hypermethylated long noncoding RNA MEG3 promotes the progression of gastric cancer
Source: Aging (Albany NY). 2019 Oct 4;11(19):8139–55. doi: 10.18632/aging.102309 (PMC6814614; doi:10.18632/aging.102309)
Supplement: Supplementary Tables [file aging-11-102309-s001.pdf]

SUPPLEMENTARY TABLES

Supplementary Table 1. Correlation between MEG3 expression and clinicopathological features in gastric cancer (GC) (n=30).

Characteristics	Total	High expression	Low expression	P value
Sex				
Male	16	7	9	0.7321
Female	14	7	7	
Age(years)				
<45	12	5	7	0.4561
≥45	18	10	8	
Extrathyroidal extension				
Yes	14	7	7	0.7321
No	16	9	7	
TNM staging				
I–II	10	7	3	0.0177*
III–IV	20	5	15	
Lymph node metastasis				
Yes	13	3	10	0.0235*
No	17	11	6	
Multicentricity				
Yes	11	5	6	0.7048
No	19	10	9	
Tumor size (cm)				
<5	11	7	4	0.0072**
≥5	19	3	16	

*P<0.05, **P<0.01

Supplementary Table 2. qRT-PCR and MSP Primer.

Gene	Primer name	Sequence
MEG3	Forward	5′- CTCCCCTTCTAGCGCTCACG-3′
	Reverse	5′- CTAGCCGCCGTCTATACTACCGGCT-3′
MEG3-M-MSP	Forward	5′- GCGAATTATTATTTATATAGCCTTC-3′
	Reverse	5′- TCACGCGCTACGAAGGGAAACG-3′
MEG3-U-MSP	Forward	5′- GTGAGGTGTTATTACCGTATAGTTTGG-3′
	Reverse	5′- TTCACACATACAGGTTCCAAACAAT-3′
miR-181a-5p	Forward	5′-ACACTCCAGCTGGGAACATTCAACGCTGTCGG-3′
	Reverse	5′-TGGTGTCGTGGAGTCG-3′
ATP4B	Forward	5′- AGGAGTTCCAGCGTTACTGC -3′
	Reverse	5′- GGTCTTGGTAGTCCGGTGTG -3′
U6	Forward	5′-GCTCGCTTCGGCAGCACAT-3′
	Reverse	5′-AAAATATGGAACGCTTCACG-3′
GAPDH	Forward	5′-GGAGCGAGATCCCTCCAAAAT-3′
	Reverse	5′-GGCTGTTGTCATACTTCTCATGG-3′
